# Supplementary material for: Public–Private Partnership: Participants’ Experiences of the Web-Based Registration-and-Management System for Patients with Hypertension and Diabetes Mellitus
Source: Healthcare (Basel). 2023 May 1;11(9):1297. doi: 10.3390/healthcare11091297 (PMC10177769; doi:10.3390/healthcare11091297)
Supplement: Supplementary file 1 [file healthcare-11-01297-s001.zip › healthcare-2292759-supplementary.pdf]

Table S1. The revised 18-question self-administered questionnaire for the web-based registration and management system for patients with hypertension and diabetes mellitus.

Component	Questions	Responses
Survey questions	After implementing the hypertension and diabetes mellitus project, do you think the number of regular visits of hypertension and diabetes mellitus patients has increased?	① Strongly increased<br>② Slightly increased<br>③ No change<br>④ Slightly decreased<br>⑤ Strongly decreased
	After implementing the hypertension and diabetes mellitus project, do you think medication compliance has improved compared to before implementing this project?	① Strongly agree<br>② Agree<br>③ Neither agree nor disagree<br>④ Disagree<br>⑤ Strongly disagree
	After implementing the hypertension and diabetes mellitus project, do you think the patients' blood pressure and glucose control are better than before?	① Strongly agree<br>② Agree<br>③ Neither agree nor disagree<br>④ Disagree<br>⑤ Strongly disagree
	After implementing the hypertension and diabetes mellitus project, do you think the patients' interest on hypertension and diabetes mellitus education materials (drug administration method, exercise, diet, etc.) has increased?	① Strongly agree<br>② Agree<br>③ Neither agree nor disagree<br>④ Disagree<br>⑤ Strongly disagree
	After implementing the hypertension and diabetes mellitus project, do you think the mutual relationship with the patients with hypertension and diabetes mellitus has improved?	① Strongly agree<br>② Agree<br>③ Neither agree nor disagree<br>④ Disagree<br>⑤ Strongly disagree
	Were the patient education materials, such as education booklets, health management diary, newsletters, etc., distributed to enrolled patients properly utilized?	① Utilized<br>② Not utilized
	After implementing the hypertension and diabetes mellitus project, what is your opinion of the said project?	① Strongly supportive<br>② Supportive<br>③ Neither supportive nor unsupportive<br>④ Unsupportive<br>⑤ Strongly unsupportive
	How do you feel about overall satisfaction after this pilot project?	① Strongly satisfied<br>② Satisfied

		③ Neither satisfied nor dissatisfied<br>④ Dissatisfied<br>⑤ Strongly dissatisfied
General characteristics of participating institutions	Age groups of the medical center directors	① 30s<br>② 40s<br>③ 50s<br>④ ≥ 60s
	Type of specialization	① Internal medicine<br>② Family medicine<br>③ Surgery<br>④ Others
	Period of medical center operation	① < 5 years<br>② 5–9 years<br>③ ≥ 10 years
	Participation period in the hypertension and diabetes mellitus project	① 0.5–1 year<br>② 1 year ≤ x < 3 years<br>③ 3 years ≤ x < 5 years<br>④ ≥ 5 years
	The average number of patients with hypertension per day	① ≤ 9 patients<br>② 10–19 patients<br>③ 20–29 patients<br>④ ≤ 30 patients
	The average number of patients with diabetes mellitus per day	① ≤ 9 patients<br>② 10–19 patients<br>③ 20–29 patients<br>④ ≤ 30 patients
	The average number of patients with hypertension and diabetes mellitus per day	① ≤ 9 patients<br>② 10–19 patients<br>③ 20–29 patients<br>④ ≤ 30 patients
	What is your job?	① Physician<br>② Nurse / Nursing Assistant<br>③ Others

Interview Questions

Table S2. Interview questions related to web-based hypertension and diabetes mellitus registration and management system participation experiences were used during the interview sessions with the participants.

Category	Questions
Introduction	Please feel free to discuss your participation experiences in the web-based registration and management system for preventing and managing hypertension and diabetes mellitus.<br>What motivated you to participate in the web-based registration and management system to prevent hypertension and diabetes mellitus?
Development	What are your participation experiences in the web-based registration and management system for preventing and managing hypertension and diabetes mellitus?<br>What did you like about your participation experiences in the web-based registration and management system for preventing and managing hypertension and diabetes mellitus? What was the problem? How did they help you?<br>Do you want to add anything to the web-based registration and management system to prevent and manage hypertension and diabetes mellitus?
Conclusion	Do you want to continue participating in the web-based registration and management system to prevent and manage hypertension and diabetes mellitus in the future?<br>Please feel free to discuss if you have any other comments on the web-based registration and management system for the prevention and management of hypertension and diabetes mellitus.
